# Audit of Cesarean Section Rates in a Tertiary Care Hospital Using the Modified Robson Classification System

**DOI:** 10.7759/cureus.110955

**Published:** 2026-06-16

**Authors:** Maria Aslam, Hasina Sadiq, Mohammad Moaz Azhar, Shaharyar Mughal, Muhammad Aqib Sattar, Hammad Ahmad Khan, Mohammad Abdul Moeed Hamid, Areej Alvi

**Affiliations:** 1 Surgical Oncology, Shaukat Khanum Memorial Cancer Hospital and Research Centre, Lahore, Lahore, PAK; 2 Obstetrics and Gynecology, KRL Hospital, Islamabad, PAK; 3 Internal Medicine, Shifa College of Medicine, Shifa Tameer-e-Millat University, Islamabad, PAK; 4 General Practice, Shifa International Hospitals Limited, Islamabad, PAK; 5 Medicine, Shifa Tameer-e-Millat University, Islamabad, PAK; 6 Basic Health Sciences, Shifa Tameer-e-Millat University, Islamabad, PAK

**Keywords:** audit cycle, cesarean section, fetal distress, modified robson classification, vbac

## Abstract

Background

The increasing rate of cesarean section (CS) has become a significant public health concern worldwide because of its association with increased maternal morbidity and healthcare burden. The Modified Robson Classification System is an internationally accepted tool for auditing and monitoring CS practices.

Objective

To evaluate CS patterns and identify the major contributors to rising CS rates in a tertiary care hospital using the Modified Robson Classification System.

Methodology

This retrospective audit was conducted in the Department of Obstetrics and Gynecology at Shifa College of Medicine from March 2023 to February 2025. A total of 493 women who underwent CS were included. Maternal demographic characteristics, obstetric history, indications for CS, and neonatal outcomes were obtained from medical records. Cases were categorized according to the Modified Robson Classification System, where Group 5C comprised women with previous cesarean scar(s) with singleton cephalic pregnancy at term, and Group 2A included nulliparous women with singleton cephalic term pregnancy undergoing induction of labor. Data were analyzed using IBM SPSS Statistics for Windows, Version 23 (Released 2016; IBM Corp., Armonk, New York, United States).

Results

The mean maternal age was 29.5 ± 5.9 years. Women with previous cesarean scars constituted the majority of cases, with Group 5C contributing 51.9% (256/493) of all CS, followed by Group 2A contributing 23.5% (116/493). Fetal distress was the leading indication for CS in 31.8% (157/493) of cases, followed by previous multiple cesarean scars in 28.0% (138/493) and refusal of trial of labor after cesarean in 18.5% (91/493). Among primigravida women, fetal distress accounted for 64.4% (85/132) of cesarean deliveries. Favorable neonatal outcomes were observed in most cases, with live births occurring in 97.6% (481/493) and NICU admissions in 2.2% (11/493) of neonates.

Conclusion

Women with previous cesarean scars and nulliparous women undergoing induction of labor were the major contributors to the overall CS rate. The Modified Robson Classification System is an effective tool for identifying high-contributing groups and guiding strategies aimed at reducing unnecessary cesarean deliveries while maintaining safe maternal and neonatal outcomes.

## Introduction

Day by day, the cesarean section (CS) rate is increasing, placing a substantial burden on healthcare systems in terms of cost, infrastructure, and resource utilization. Although several contributing factors have been identified, the primary drivers of this rising trend remain incompletely understood. To facilitate standardized assessment and monitoring of cesarean deliveries, the Robson Ten Group Classification System was introduced in 2001 and subsequently endorsed by the World Health Organization and International Federation of Gynecology and Obstetrics in 2015 as a women-centered classification applicable across different healthcare settings [1]. A French study reported that the CS rate increased from approximately 12% in 2015 to 21.1% in 2020, with higher rates observed among Asian populations compared to European and Western countries [2]. The Robson classification categorizes women into 10 distinct groups based on obstetric characteristics, parity, fetal presentation, gestational age, onset of labor, and previous cesarean delivery. The WHO recommends an optimal CS rate of 10-15%, as rates exceeding this threshold have not shown additional improvement in maternal or neonatal outcomes and may instead be associated with adverse consequences [2].

The Society of Obstetricians and Gynaecologists of Canada (SOGC) later proposed a modified Robson classification system that further subdivided the original 10 groups into categories based on cesarean delivery before labor, after induction of labor (IOL), and following spontaneous onset of labor [3]. An Australian study evaluating the modified Robson criteria demonstrated a marked increase in cesarean deliveries from 18% to 35% across tertiary maternity centers, emphasizing the utility of the classification system in institutional audits and quality improvement initiatives [4].

According to the National Health Service, a clinical audit is a quality improvement process that systematically reviews patient care against established standards to improve clinical outcomes [5]. Clinical audit cycles allow comparison of existing practices with evidence-based standards, facilitate the identification of deficiencies, and support the implementation of corrective measures to optimize patient management and outcomes [6].

Although cesarean delivery is a life-saving intervention when medically indicated, unnecessary procedures are associated with increased maternal morbidity, prolonged hospital stay, higher healthcare expenditure, postoperative complications, hemorrhage, infections, neonatal intensive care unit (NICU) admissions, and psychological consequences [7]. Previous studies have identified several factors contributing to rising cesarean rates, including inadequate fetal monitoring facilities, reduced expertise in instrumental vaginal deliveries, institutional protocols, medico-legal concerns, and financial reimbursement policies [8-10].

CS rates vary considerably among institutions because of differences in available resources, obstetric expertise, institutional practices, patient risk profiles, and referral patterns from peripheral healthcare facilities. High CS rates have been reported in several Asian countries with high Human Development Index (HDI) scores, including Sri Lanka (33%), China (47%), and Thailand (39.4%) [9]. Similarly, Pakistan has experienced a substantial increase in cesarean births over the past two decades, with national rates rising from 3.2% in 1990 to 19.7% in 2018 [10-13]. Local studies conducted in tertiary care hospitals in Pakistan using the Modified Robson Classification have highlighted the significant contribution of previous CS and induced nulliparous pregnancies to the rising CS rate [14-16]. Therefore, the purpose of conducting this audit was not only to identify the obstetric groups contributing most to the CS rate but also to determine potentially modifiable factors that could be addressed through evidence-based interventions. By identifying high-contributing groups according to the Modified Robson Classification System, such as women with previous CS and nulliparous women undergoing IOL, the audit provides a framework for targeted strategies, including promotion of vaginal birth after cesarean (VBAC), optimization of induction protocols, improvement in intrapartum fetal monitoring practices, and regular clinical audit cycles. These measures may help reduce unnecessary cesarean deliveries while maintaining maternal and neonatal safety.

## Materials and methods

Aim

The audit was designed to determine the CS rate at the tertiary care hospital.

Objectives

To identify the factors leading to an increased rate of operative deliveries after the implementation of the Modified Robson Classification and to highlight those groups in which changes could be made to improve the CS rate.

Standard

The SOGC proposed in 2012 that the original 10-group Robson criteria be modified by adding three subgroups to each major category of women who underwent CS after spontaneous onset of labor, after IOL, and before labor. Therefore, the Modified Robson Criteria were used as the standard for this audit (Table 1).

**Table 1:** Modified Robson Classification Table This table has been reproduced with permission from the original publisher [1].

Group	Classification	Subcategories
Group 1	Nullipara, singleton, cephalic, >37 weeks, spontaneous labour	
Group 2	Nullipara, singleton, cephalic, >37 weeks	A. Induced B. Cesarean section before labour
Group 3	Multipara, singleton, cephalic, >37 weeks, spontaneous onset of labour	
Group 4	Multipara, singleton, cephalic, >37 weeks	A. Induced B. Cesarean section before labour
Group 5	Previous cesarean section, singleton, cephalic, >37 weeks	A. Spontaneous labour B. Induced C. Cesarean section before labour
Group 6	All nulliparous breeches	A. Spontaneous labour B. Induced C. Cesarean section before labour
Group 7	All multiparous breeches (including previous cesarean sections)	A. Spontaneous labour B. Induced C. Cesarean section before labour
Group 8	All multiple pregnancies	A. Spontaneous labour B. Induced C. Cesarean section before labour
Group 9	All abnormal lies (including previous cesarean sections but excluding breech)	A. Spontaneous labour B. Induced C. Cesarean section before labour
Group 10	All singleton, cephalic, <36 weeks (including previous cesarean section)	A. Spontaneous labour B. Induced C. Cesarean section before labour

This retrospective audit was carried out in the Department of Obstetrics and Gynecology at Shifa College of Medicine, Islamabad. Relevant institutional ethical committee approval was obtained. Since this was a retrospective clinical audit, all CS cases performed between March 2023 and February 2025 that met the inclusion criteria were included. Therefore, no sample size calculation was required, and a census sampling technique was employed, resulting in a total sample of 493 patients. The confidential medical record (CMR) files of patients were obtained from the medical records department. Informed consent from patients was not required due to the retrospective nature of the audit.

All women who underwent CS during the study period were included. Pregnancies terminated due to fetal abnormalities were excluded from the study. The audit team consisted of two senior registrars and one Assistant Professor from Shifa College of Medicine, and the audit was supervised by the Professor of the institution.

Data were recorded on a specially designed proforma that included maternal baseline characteristics and obstetric history, including maternal age, parity, number of previous CS, number of fetuses in the current pregnancy (singleton or multiple), gestational age at delivery, fetal lie and presentation (cephalic, breech, or transverse), onset of labor (spontaneous or induced), type of procedure performed (emergency or elective CS), and associated medical or surgical comorbidities. Births were classified according to the Modified Robson Criteria and further subcategorized where required. The indications for CS were also specified, including placenta previa and placenta accreta spectrum, fetal distress, failed induction, non-progress of labor, oligohydramnios, and other miscellaneous indications.

All cases were reviewed, investigated, and classified accordingly. Data were analyzed using IBM SPSS Statistics for Windows, Version 23 (Released 2016; IBM Corp., Armonk, New York, United States). The total number of deliveries, total number of CS, and overall CS rate were calculated for each category and subcategory according to the Modified Robson 10-group classification system, along with their contribution to the overall CS rate. Qualitative variables were presented as frequencies and percentages, whereas quantitative variables were expressed as mean ± standard deviation. Frequencies and proportions were reported with 95% confidence intervals where appropriate.

## Results

The study included a total of 493 patients, with a mean age of 29.5 ± 5.9 years, highlighting a relatively young cohort. In terms of parity, 177 (35.9%) patients were primary gravida (para 0), indicating their first pregnancy, while 129 (26.2%) were para 1, 112 (22.7%) were para-2, 56 (11.4%) were para 3, and 19 (3.9%) had four or more previous pregnancies. Examining previous modes of delivery, 177 (35.9%) patients had no history of prior delivery, 41 (8.3%) had previously delivered via spontaneous vaginal delivery (SVD), and 275 (55.8%) had undergone a lower-segment cesarean section (LSCS). This distribution provides insights into both the patients' reproductive histories and their prior delivery experiences, as shown in Table 2.

**Table 2:** Baseline Characteristics of Patients SVD: spontaneous vaginal delivery; LSCS: lower-segment cesarean section

Characteristics	Value
Age of the patients (years)	29.50 ± 5.90
Parity status of the patients	
Para 0 (Primary gravida)	177 (35.9%)
Para 1	129 (26.2%)
Para 2	112 (22.7%)
Para 3	56 (11.4%)
Para 4 and above	19 (3.9%)
Previous mode of delivery	
Previous no delivery	177 (35.9%)
SVD	41 (8.3%)
LSCS	275 (55.8%)
Total	493 (100.0%)

The study classified 493 patients based on Modified Robson’s Group, with the highest proportion in Group 5C at 256 (51.9%), followed by Group 2A at 116 (23.5%). Other groups included Group 2B with 28 (5.7%), Group 4A with 30 (6.1%), Group 1 with 17 (3.4%), and smaller percentages across groups such as Group 5A with 12 (2.4%) and Group 8C with 11 (2.2%). The least represented groups were Group 7C, Group 8A, and Group 10A, each at 1 (0.2%). Indications for CS included fetal distress in 157 (31.8%) cases, previous multiple cesareans in 138 (28.0%), and refusal of trial after a previous cesarean in 91 (18.5%). Additional indications were elective cesarean (refusal of trial) at 37 (7.5%), non-progress of labor at 31 (6.3%), failed IOL at nine (1.8%), severe oligohydramnios at eight (1.6%), maternal conditions (hypertension and diabetes) at seven (1.4%), and twin pregnancies refusing trial at seven (1.4%). Less frequent reasons included placenta previa or accreta spectrum at four (0.8%) and malpresentation at four (0.8%). The baby status showed 481 (97.6%) live births, one (0.2%) stillbirth, and 11 (2.2%) babies requiring NICU admission, as elaborated in Table 3.

**Table 3:** Distribution of Modified Robson Classification Groups, Indications for Cesarean Section, and Baby Status * Refused trial refers to patients with a previous cesarean section who declined a trial of labor after cesarean (TOLAC) and opted for repeat cesarean delivery. ** Previous 2/3/4 scar refers to patients with a history of two, three, or four previous cesarean section scars, respectively. IOL: induction of labor; LSCS: lower-segment cesarean section; NICU: neonatal intensive care unit

Characteristics	Frequency	Percentage (%)
Modified Robson’s Group		
Group 1	17	3.4
Group 2A	116	23.5
Group 2B	28	5.7
Group 3	4	0.8
Group 4A	30	6.1
Group 4B	7	1.4
Group 5A	12	2.4
Group 5C	256	51.9
Group 6A	4	0.8
Group 6C	5	1.0
Group 7C	1	0.2
Group 8A	1	0.2
Group 8C	11	2.2
Group 10A	1	0.2
Indications for C-section		
Fetal distress	157	31.8
Non progress of labour	31	6.3
Failed IOL	9	1.8
Maternal pre-existing hypertension and diabetes	7	1.4
Placenta previa and accreta spectrum	4	0.8
Elective LSCS (refused trial)*	37	7.5
Previous LSCS refused TOLAC	91	18.5
Severe oligohydramnios	8	1.6
Previous 2/3/4 scar**	138	28.0
Twin pregnancy refused trial	7	1.4
Mal-presentation	4	0.8
Baby status		
Alive	481	97.6
Stillbirth	1	0.2
NICU stay	11	2.2
Total	493	100.0

Among the 177 primigravida patients undergoing CS, the most common indication was fetal distress, accounting for 114 (64.4%) cases. Other indications included non-progress of labor in 19 (10.7%) patients, elective LSCS due to refusal of trial in 20 (11.3%), and failed IOL in eight (4.5%). Less frequent reasons were severe oligohydramnios in six (3.4%) cases, twin pregnancy refusal of trial in four (2.3%), and both placenta previa/accreta spectrum and malpresentation, each at three (1.7%), as shown in Table 4.

**Table 4:** Distribution of Indications for Cesarean Section Among Primigravid Women IOL: induction of labor; LSCS: lower-segment cesarean section

Indications	Frequency	Percentage (%)
Fetal distress	114	64.4
Non-progress of labour	19	10.7
Failed IOL	8	4.5
Placenta previa and accreta spectrum	3	1.7
Elective LSCS (refused trial)	20	11.3
Severe oligohydramnios	6	3.4
Twin pregnancy refused trial	4	2.3
Malpresentation	3	1.7
Total	177	100.0

Table 5 summarizes the distribution of indications for CS among patients with one previous scar, a total of 256 cases. The most common indication was refusal of a trial of labor after cesarean (TOLAC) following a prior lower-segment C-section (LSCS), observed in 147 (57.4%) cases, followed by elective LSCS without trial in 88 (34.4%) cases. Other indications included fetal distress in nine (3.5%) cases, maternal pre-existing hypertension and diabetes in four (1.6%) cases, and severe oligohydramnios in three (1.2%) cases. Less frequent reasons were twin pregnancy with refusal of trial in two (0.8%) cases, and placenta previa/accreta spectrum, non-progression of labor, and malpresentation, each reported in one (0.4%) case. The total sample comprised 256 (100.0%) cases.

**Table 5:** Distribution of Indications of C-section Among Patients With Previous One Cesarean Section Scar LSCS: lower-segment cesarean section; TOLAC: trial of labor after cesarean

Indications	Frequency	Percentage(%)
Fetal distress	9	3.5
Non-progress of labour	1	0.4
Maternal pre-existing hypertension and diabetes	4	1.6
Placenta previa and accreta spectrum	1	0.4
Elective LSCS (refused trial)	88	34.4
Previous LSCS refused TOLAC	147	57.4
Severe oligohydramnios	3	1.2
Twin pregnancy refused trial	2	0.8
Malpresentation	1	0.4
Total	256	100.0

## Discussion

The present audit evaluated CS patterns in a tertiary care hospital using the modified Robson classification system. The findings demonstrated that Group 5C, comprising women with multiple previous cesarean scars, was the largest contributor to the overall CS rate, followed by Group 2A, which included nulliparous women undergoing IOL. These findings are consistent with international literature and highlight the importance of identifying specific obstetric populations responsible for the increasing trend of cesarean deliveries [4,11].

In the current study, more than half of all CS were performed in women belonging to Group 5C. Similar observations have been reported in studies from Pakistan, Australia, and other international settings, where women with a previous CS represent the major contributor to institutional CS rates [2-10]. The increasing number of repeat cesarean deliveries is largely associated with reduced acceptance of VBAC, fear of uterine rupture, medico-legal concerns, and maternal preference for elective repeat surgery. In the present study, refusal of TOLAC was a major indication for repeat CS. Evidence suggests that appropriately selected women undergoing VBAC have good maternal and neonatal outcomes, emphasizing the importance of adequate antenatal counseling and institutional VBAC protocols [12].

Group 2A was the second largest contributor to the overall CS rate. This group consisted of nulliparous women who underwent IOL and subsequently required cesarean delivery. Previous studies have similarly demonstrated higher operative delivery rates among induced primigravida women, particularly in cases with unfavorable cervical status or prolonged labor [1-4]. In the present study, fetal distress was the leading indication for CS among primigravida patients, accounting for approximately two-thirds of cases. Comparable findings have been reported in other Robson-based audits, where abnormal fetal heart rate interpretation contributed significantly to emergency cesarean deliveries [5].

The high proportion of CS performed for fetal distress observed in this study may reflect variability in intrapartum fetal monitoring practices. Continuous cardiotocography, although widely used, has been associated with increased CS rates because of its limited specificity in predicting true fetal compromise. Previous literature suggests that standardized fetal monitoring protocols, regular staff training, and appropriate use of adjunctive fetal assessment methods can reduce unnecessary operative interventions without compromising neonatal safety [5,11]. Therefore, improving labor room monitoring practices and strengthening interpretation skills may help reduce avoidable cesarean deliveries in this setting.

Another notable finding was the substantial number of elective CS performed because of maternal refusal of labor trials. Maternal preference for cesarean delivery has become an increasingly recognized factor contributing to rising CS rates worldwide. Fear of labor pain, anxiety related to previous birth experiences, concerns regarding neonatal outcomes, and inadequate counseling may influence patient decisions regarding mode of delivery [11]. Structured antenatal counseling programs and shared decision-making approaches may improve maternal confidence in vaginal birth and enhance acceptance of TOLAC where clinically appropriate.

Neonatal outcomes in the present audit were generally favorable, with a high proportion of live births and a relatively low NICU admission rate. Although these findings suggest satisfactory short-term neonatal outcomes, repeated CS remain associated with significant long-term maternal risks, including placenta accreta spectrum, surgical adhesions, hemorrhage, and operative complications in subsequent pregnancies. Previous studies have demonstrated that maternal morbidity increases progressively with each additional cesarean delivery, particularly among women with multiple uterine scars [13]. This is especially relevant in the current study, where Group 5C represented the largest proportion of cesarean deliveries.

The modified Robson classification system proved to be an effective and practical auditing tool in the present study. Its standardized structure allows comparison of CS trends across institutions and helps identify target groups requiring intervention. The classification system has been endorsed by the World Health Organization and International Federation of Gynecology and Obstetrics as a reliable method for monitoring and evaluating institutional CS practices [5,9]. Regular clinical audit using the Robson classification, combined with evidence-based labor management protocols and promotion of VBAC, may help optimize CS rates while maintaining favorable maternal and neonatal outcomes.

Strengths

The present study has several notable strengths. First, it utilized the Modified Robson Classification System, an internationally recognized and standardized framework endorsed by the World Health Organization and International Federation of Gynecology and Obstetrics, allowing meaningful comparison with national and international data. Second, the study included a relatively large sample of 493 cesarean deliveries collected over a 24-month period, reducing the risk of seasonal variation and providing a comprehensive overview of institutional practice patterns. Third, subgroup analyses according to parity and previous CS status provided additional clinical insight into the major contributors to operative deliveries. These strengths enhance the clinical relevance of the findings and support the identification of target groups for future quality-improvement interventions.

Limitations

This study has several limitations. First, it was a retrospective single-center study, which may limit the generalizability of the findings to other institutions and healthcare settings. Second, the dataset included only women who underwent CS and did not include total institutional deliveries, SVDs, or instrumental vaginal deliveries. Consequently, the overall institutional CS rate and the complete set of standard Robson indicators could not be calculated.

Third, assignment of cases to Modified Robson groups was based on retrospective review of medical records, and formal inter-rater reliability assessment was not performed. Therefore, the possibility of classification bias cannot be completely excluded. Fourth, severe maternal morbidity, postoperative complications, and long-term maternal and neonatal outcomes were not evaluated, limiting assessment of the broader clinical impact of cesarean delivery, particularly among women in Group 5C.

Fifth, factors influencing maternal preference for cesarean delivery and refusal of TOLAC could not be explored in detail because of limitations inherent to retrospective record review. Finally, although the study included 24 months of consecutive data, temporal trends in CS patterns were not analyzed, representing a missed opportunity for further evaluation of changes over time.

Furthermore, while the study utilized an audit framework based on the Modified Robson Classification System, it did not include implementation of corrective interventions or a re-audit cycle. Therefore, the findings should be interpreted as a descriptive retrospective audit rather than a complete clinical audit cycle.

## Conclusions

The present audit demonstrated that women with previous cesarean scars, particularly those classified in Modified Robson Group 5C, were the predominant contributors to the overall CS rate, followed by nulliparous women undergoing IOL in Group 2A. Fetal distress and refusal of TOLAC were the leading indications for operative delivery. The modified Robson classification system provided an effective framework for identifying target groups contributing disproportionately to cesarean births. Strengthening VBAC counseling, improving intrapartum fetal monitoring interpretation, promoting evidence-based induction protocols, and implementing regular clinical audits may help reduce unnecessary cesarean deliveries while maintaining safe maternal and neonatal outcomes.
